# Hydrogen gas inhalation improves delayed brain injury by alleviating early brain injury after experimental subarachnoid hemorrhage

**DOI:** 10.1038/s41598-020-69028-5

**Published:** 2020-07-23

**Authors:** Kosuke Kumagai, Terushige Toyooka, Satoru Takeuchi, Naoki Otani, Kojiro Wada, Arata Tomiyama, Kentaro Mori

**Affiliations:** 1 Department of Neurosurgery, National Defense Medical College, 3-2 Namiki, Tokorozawa, Saitama 359-8513 Japan; 2 Department of Neurosurgery, Tokyo General Hospital, Tokyo, Japan

**Keywords:** Diseases, Molecular medicine, Neurology

## Abstract

Molecular hydrogen (H_2_) protect neurons against reactive oxygen species and ameliorates early brain injury (EBI) after subarachnoid hemorrhage (SAH). This study investigated the effect of H_2_ on delayed brain injury (DBI) using the rat SAH + unilateral common carotid artery occlusion (UCCAO) model with the endovascular perforation method. 1.3% H_2_ gas (1.3% hydrogen premixed with 30% oxygen and balanced nitrogen) inhalation was performed on days 0 and 1, starting from anesthesia induction and continuing for 2 h on day 0, and starting from anesthesia induction and continuing for 30 min on day 1. EBI was assessed on the basis of brain edema, expression of S100 calcium-binding protein B (S100B), and phosphorylation of C-Jun N-terminal kinase on day 2, and neurological deficits on day 3. Reactive astrogliosis and severity of cerebral vasospasm (CV) were assessed on days 3 and 7. DBI was assessed on the basis of neurological deficits and neuronal cell death on day 7. EBI, reactive astrogliosis, and DBI were ameliorated in the H_2_ group compared with the control group. CV showed no significant improvement between the control and H_2_ groups. This study demonstrated that H_2_ gas inhalation ameliorated DBI by reducing EBI without improving CV in the rat SAH + UCCAO model.

## Introduction

Aneurysmal subarachnoid hemorrhage (SAH) is still a devastating disease^1^. The pathophysiological changes following SAH are commonly divided into early brain injury (EBI) and delayed brain injury (DBI). The term delayed cerebral ischemia (DCI) is more commonly used than DBI for describing critical events arising in the late phase of SAH^2^. DCI occurs in about 30% of surviving SAH patients and is the most important cause of morbidity and mortality after SAH. Recently, late phase brain damage was reported to result from the interaction of multiple pathological pathways including edema formation and neuro-inflammatory responses with or without ischemia^3^. Therefore, this study has used the term DBI instead of DCI. Recent studies have demonstrated that cerebral vasospasm (CV) does not necessarily lead to DBI and that DBI can occur in the absence of CV^4^. EBI occurring within 72 h after SAH is increasingly recognized as important in DBI. Recent more effective treatment of EBI may reduce the severity of DBI regardless of the occurrence of CV^3,5^.

Molecular hydrogen (H_2_) is known to protect neurons against reactive oxygen species (ROS) and has ameliorative effects on ischemia/reperfusion injury with few side effects^6^. H_2_ gas or H_2_-rich saline also has beneficial effects against EBI after SAH^7,8^. H_2_ suppresses expression of S100 calcium-binding protein B (S100B), phosphorylation of C-Jun N-terminal kinase (JNK), and reactive astrogliosis through reduction of ROS and lower neuronal cell damage in several rat disease models except for SAH^9–11^. However, the effects of H_2_ on DBI remain unclear.

Recently, we developed and reported a new modified rat endovascular perforation (EVP) SAH model, the SAH + unilateral common carotid artery occlusion (UCCAO) model^12^. In this SAH + UCCAO model, mild EVP SAH-induced UCCAO was combined with early cerebral hypoperfusion begun 24 h after SAH induction to mimic the clinical course of early cerebral hypoperfusion after SAH. This model is useful to elucidate the pathophysiological mechanisms of EBI, CV, and DBI without high mortality^12^.

This study investigated the effect of H_2_ gas inhalation on EBI, CV, and subsequent DBI using the SAH + UCCAO model.

## Materials and methods

### Animals

All experiments and methods were carried out in accordance with relevant guidelines and regulations. All experimental procedures used in this study were approved by the Institutional Animal Care and Use Committee (IACUC) at National Defense Medical College. A total of 101 adult male Sprague–Dawley rats (Japan SLC Inc., Shizuoka, Japan), weighing 300–400 g, were housed in a vivarium with a 12-h light/dark cycle and free access to food and water. Rectal temperature was maintained at close to 37.0 °C with a heating pad and/or heating lamp during the experiments.

### SAH + UCCAO model

The EVP model of SAH in rats was described previously^13^. The rat SAH + UCCAO modification of the EVP model was published recently^12^. Briefly, general anesthesia was induced with 3% isoflurane in a mixture of 70% nitrogen and 30% oxygen (70%/30% gas) in the sham and control groups. In the H_2_ group, 1.3% H_2_ gas (1.3% hydrogen premixed with 30% oxygen and balanced nitrogen) was used starting from anesthesia induction instead of the 70%/30% gas. Buprenorphine (0.01 mg/kg) was injected intraperitoneally. An arterial line was inserted into the proximal segment of the tail artery and connected to a blood-pressure transducer. Tracheal intubation was performed after administration of vecuronium bromide (0.15 mg/kg). The rats were mechanically ventilated and anesthesia was maintained with 1.5–2% isoflurane. Arterial blood gases (pH, PaCO_2_, and PaO_2_) were measured after intubation. The head was positioned in a stereotactic frame and the lambda was exposed with a 1.5-cm midline dorsal incision. Continuous monitoring of intracranial pressure (ICP) was performed by placing an ICP probe (Codman, Raynham, Massachusetts, USA) in the epidural space through a small right parietal craniotomy. The left external carotid artery was isolated through a midline incision in the necks. A sharpened 6-0 Prolene suture (Ethicon, USA) was inserted from the external carotid artery through the internal carotid artery to perforate the intracranial bifurcation and induce SAH, then the suture was removed. Mean arterial blood pressure (MABP) and ICP were recorded continuously during the initial 30 min of the experiment with a data acquisition device (PowerLab, ADInstruments, Spechbach, Germany). At the end of the surgery, the wounds were closed. Sham-operated rats underwent the same procedures except for perforation. All rats were ventilated until recovery from anesthesia. After extubation, the rats were returned to the cages with free access to food and water.

Rats excluding dead rats (neurological score of 3) and rats with neurological score between 4 and 14 in the sham, control, and H_2_ groups at day 1 after SAH were anesthetized with a face mask using 1.5–2% isoflurane in 70%/30% gas following induction of anesthesia with 3% isoflurane, or 1.3% H_2_ gas instead of 70%/30% gas in the H_2_ group. An arterial line was placed at the base of the tail artery. Arterial blood gases (pH, PaCO_2_, and PaO_2_) were measured before UCCAO, and MABP was recorded continuously for 3 min before and after UCCAO. The midline neck incision was reopened, and the left carotid artery (the left external carotid artery had already been ligated at day 0) was carefully exposed and ligated using the two 4-0 silk sutures previously inserted under the left carotid artery on day 0. Then, the left carotid artery was cut between these two ligations. At the end of the surgery, the wounds were closed and the rats were returned to the cages with free access to food and water.

### H_2_ gas

1.3% H_2_ gas (1.3% hydrogen premixed with 30% oxygen and balanced nitrogen) was purchased from the manufacturer (Saisan Inc., Saitama, Japan)^14^. 1.3% H_2_ gas inhalation was administered twice on days 0 and 1, starting at anesthesia induction and continuing for 2 h on day 0, and starting at anesthesia induction and continuing for 30 min on day 1.

### Experimental groups

**Figure 1:**
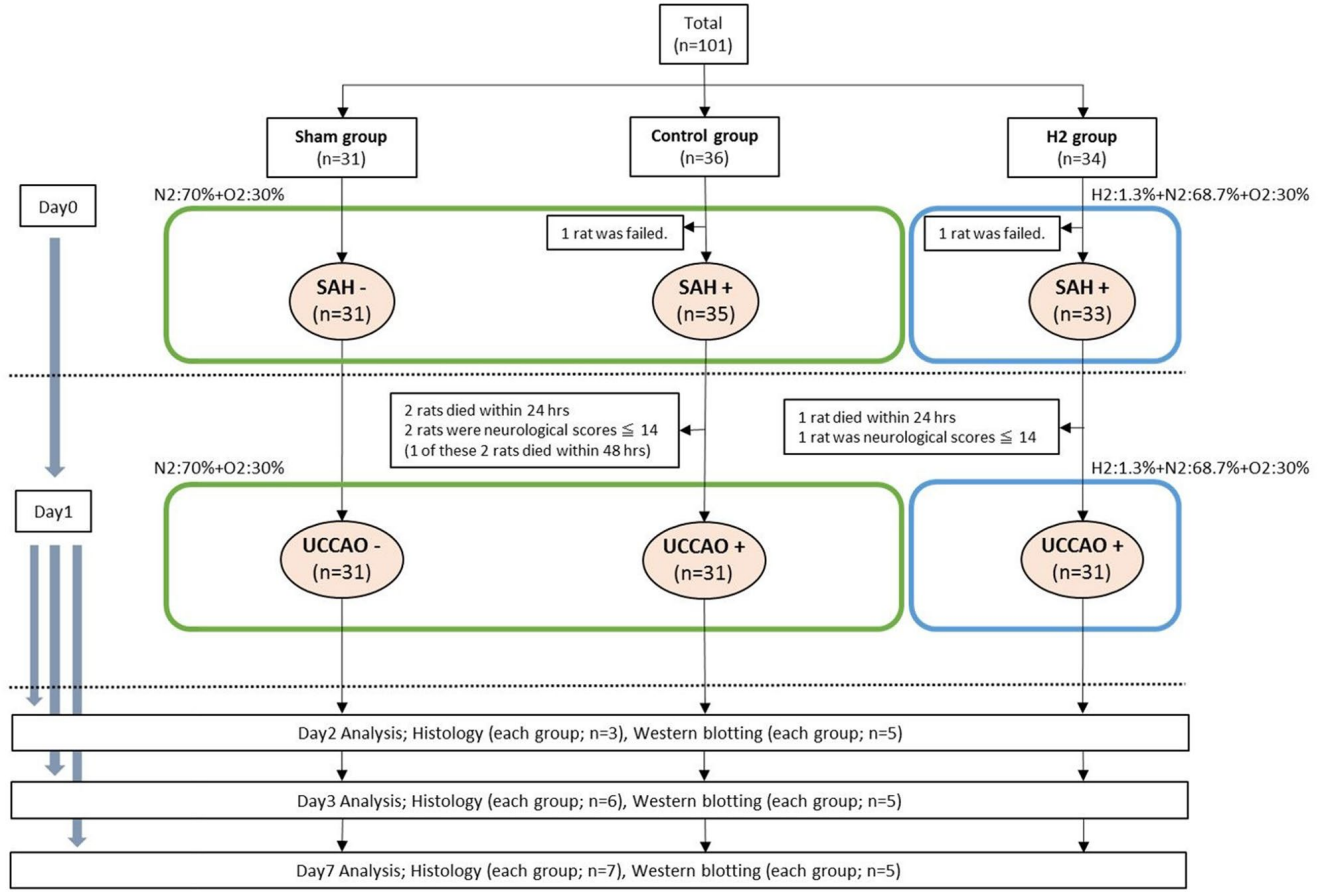
Flow chart of the experimental protocol: total sample size, n = 101. *SAH* subarachnoid hemorrhage, *UCCAO* unilateral common carotid artery occlusion, *H*_2_ hydrogen, *N*_2_ nitrogen, *O*_2_ oxygen.

The rats were randomly divided into the sham group (n = 31), control group (n = 36), and H_2_ group (n = 34). No ICP change occurred after perforation of the internal carotid artery bifurcation in 1 rat in each of the control and H_2_ groups, and these rats were excluded. UCCAO was then performed in the SAH rats with neurological score of 15 or higher under 70%/30% gas in the control group (n = 31) and under 1.3% H_2_ gas in the H_2_ group (n = 31) (Fig. 1) as described previously^12^.

### Mortality rate, neurological deficits, and body weight

Mortality rate was calculated within 24 h and until day 7 after SAH induction. Body weight was measured once a day on days 0 (baseline), 1 (after exclusion), 2, 3, and 7 (n = 31 per group on days 1 and 2; n = 23 per group on day 3; n = 12 per group on day 7). Neurological deficits were evaluated on day 1 before exclusion (n = 31 in sham group, n = 35 in control group, and n = 33 in H_2_ group) and at sacrifice (n = 8 per group on day 2; n = 11 per group on day 3; n = 12 per group on day 7) by an independent observer according to the modified Garcia scoring system^15,16^. Briefly, the evaluation indexes were as follows: spontaneous activity (0–3 points); symmetry in movement of the four limbs (0–3 points); forepaw outstretching (0–3 points); climbing (1–3 points); body proprioception (1–3 points); and response to vibrissae touch (1–3 points). Animals were given a score of 3–18 (higher scores indicate greater function).

### Specimen preparation

Specimens for histological analysis with hematoxylin and eosin staining, Nissl staining, and immunohistochemistry were prepared as follows. Animals were perfused transcardially with normal saline followed by 4% phosphate-buffered paraformaldehyde under deep anesthesia induced by inhalation of 5% isoflurane (n = 3 per group on day 2; n = 6 per group on day 3; n = 7 per group on day 7). Brains were excised and postfixed in the same fixative overnight, then cut into several blocks including the hippocampus. The tissue blocks were routinely embedded in paraffin and 5-µm thick sections were cut using a microtome. Specimens for measurement of brain water content on day 2 and immunoblot analysis on days 2, 3, and 7 (n = 5 per group per time point) were prepared as follows. Animals were perfused transcardially with ice cold saline under deep anesthesia induced by inhalation with 5% isoflurane. Brains were immediately removed and the tissues in the left temporal cortex (ipsilateral to SAH induction) were dissected and stored at – 196 °C in liquid nitrogen. The left frontal hemisphere was divided from the removed brain on day 2 for measurement of brain water content.

### Brain water content

Brain water content was assayed on day 2 after SAH in animals from the Western blot analysis group. Briefly, the left frontal hemisphere was weighed immediately after division from the brain (wet weight). After dehydration at 105 °C for 72 h, the sample was weighed again (dry weight). The brain water content was calculated using the following formula: brain water content (%) = (wet weight − dry weight)/wet weight × 100.

### Western blot analysis

The rat brain samples were treated in lysis buffer (0.5 mol/L Tris–HCl [pH 6.8], 10% glycerol, 2% sodium dodecyl sulfate) followed by sonication. Immunoblot analysis of the obtained brain tissue lysates was performed as described previously^17^ using the following primary antibodies: mouse monoclonal anti-glial fibrillary acidic protein (GFAP) (Cell Signaling Technology, #3670; 1:1,000); rabbit monoclonal anti-S100B (GeneTex, #GTX129573; 1:1,000); rabbit monoclonal anti-phosphorylated JNK (p-JNK) (Cell Signaling Technology, #4668; 1:1,000); and rabbit monoclonal anti-glyceraldehyde-3-phosphate dehydrogenase (Cell Signaling Technology, #8884; 1:1,000). The final chemiluminescence images of the polyvinylidene difluoride membranes were captured using an Amersham Imager 600 (GE Healthcare Life Sciences).

### Histological examination

#### Assessment of CV

The inner diameter and wall thickness of the distal anterior cerebral artery (ACA) were measured on specimen slices after hematoxylin and eosin staining^18^. Cross-sections of the region from the optic chiasma level to the hippocampal level were prepared on days 3 and 7, respectively. The inner diameter was calculated as the average of the maximum and minimum diameter, and the wall thickness was calculated as the average at the 3, 6, 9, and 12 o’clock positions by two independent observers using an all-in-one fluorescence microscope (BZ-X700, KEYENCE Co., Osaka, Japan).

#### Assessment of neuronal cell death

Quantitative assessment counted the numbers of vital and non-vital neuronal cells in the left temporal cortex and the left dentate gyrus (DG) (ipsilateral to SAH induction) on day 7. Neurons were classified as non-vital if exhibiting shrinkage of dark purple stains, disappearance of the nucleolus, and appearance of vacuoles around the cells^14^. The regions of interest in the cortex and DG were 0.25 mm^2^ and 0.04 mm^2^, respectively. The cells were counted by two independent observers using the BZ-X700 fluorescence microscope. Brain sections including the hippocampus were dried at 37 °C for 30 min and hydrated in 0.1% cresyl violet for 5 min for Nissl staining. After rinsing with water, sections were dehydrated in increasing concentrations of ethanol and cleared of xylenes, then mounted with permount reagent, coverslipped, and observed under a light microscope.

#### Immunohistochemistry of GFAP

Sections of 5 μm thickness were cut from the paraffin-embedded slices including the hippocampus obtained on days 3 and 7. After treatment with blocking serum, the sections were incubated with mouse monoclonal anti-GFAP (Cell Signaling Technology, #3670; 1:50) overnight at 4 °C. Histofine Simple Stain MAX PO (Nichirei Biosciences Inc., Tokyo, Japan) was used as the secondary antibody followed by visualization with 3,3′-diaminobenzidine (Muto Pure Chemicals Co., Ltd., Tokyo, Japan) as a chromogen. After the nucleus was counterstained with Meyer’s hematoxylin, the slides were dehydrated and mounted. Sections were observed using the BZ-X700 fluorescence microscope.

#### Immunofluorescence staining

A series of 5 μm slices were cut from the paraffin-embedded slices including the hippocampus obtained on day 2. Double immunofluorescence staining was performed as previously described^19^. Sections were incubated with primary antibodies overnight in a humidified chamber at 4 °C. Primary antibodies were rabbit monoclonal anti-p-JNK (Abcam, ab4821; 1:200), anti-neuronal nuclear antigen (Millipore, MAB377; 1:200), mouse monoclonal anti-GFAP (Cell Signaling Technology, #3,670; 1:50), and anti-Iba-1 (Abcam, ab1690; 1:200). For fluorescence staining, sections were incubated with secondary antibodies using Alexa flour-conjugated goat anti-mouse (Abcam, ab150113; 1:200) and goat anti-rabbit Cy3 (Jackson ImmunoResearch, 111-165-144; 1:200). Sections were examined using the BZ-X700 fluorescence microscope.

#### Quantification and statistical analysis

Prism 8 (GraphPad) was used for statistical analysis. Data are expressed as mean ± standard deviation. Mortality rate was analyzed by the Fischer exact test. Two-way analysis of variance (ANOVA) with repeated measures was used to compare time course of ICP and cerebral perfusion pressure (CPP) between the three groups followed by the Tukey–Kramer multiple comparison procedure. All other values were analyzed by one-way ANOVA followed by the Tukey–Kramer multiple comparison procedure. Statistical significance was considered at *P* < 0.05.

## Results

The SAH + UCCAO model was used to assess the efficacy of H_2_ gas inhalation in EBI, reactive astrogliosis, CV, and DBI. EBI was evaluated based on neurological deficits, brain edema, and expression of S100B and p-JNK at day 2. Reactive astrogliosis was measured as up-regulation of GFAP at days 3 and 7. Severity of CV was assessed in the distal ACA. DBI was evaluated based on neurological deficits and neuronal cell death at day 7.

### Mortality rate and exclusion

The mortality rate within 24 h was 5.7% (2/35 rats) and 3.0% (1/33 rats) in the control and H_2_ groups, respectively, with no significant difference (*P* > 0.05). The control group included 2 rats with neurological score of less than 14 on day 1. One rat died within 48 h and 1 rat survived until day 7. The H_2_ group contained 1 rat with neurological score of less than 14 on day 1, which survived until day 7. No rat died in the sham group, or in both the control and H_2_ groups after addition of UCCAO.

### Physiological parameters

No significant changes in the physiological parameters of baseline body weight on day 0, and MABP and blood gases before the procedures on days 0 (n = 31 in sham group, n = 35 in control group, and n = 33 in H_2_ group; *P* > 0.05, respectively) and 1 (n = 31 per group; *P* > 0.05, respectively) occurred in the three groups.

### ICP, MABP, and CPP

**Figure 2:**
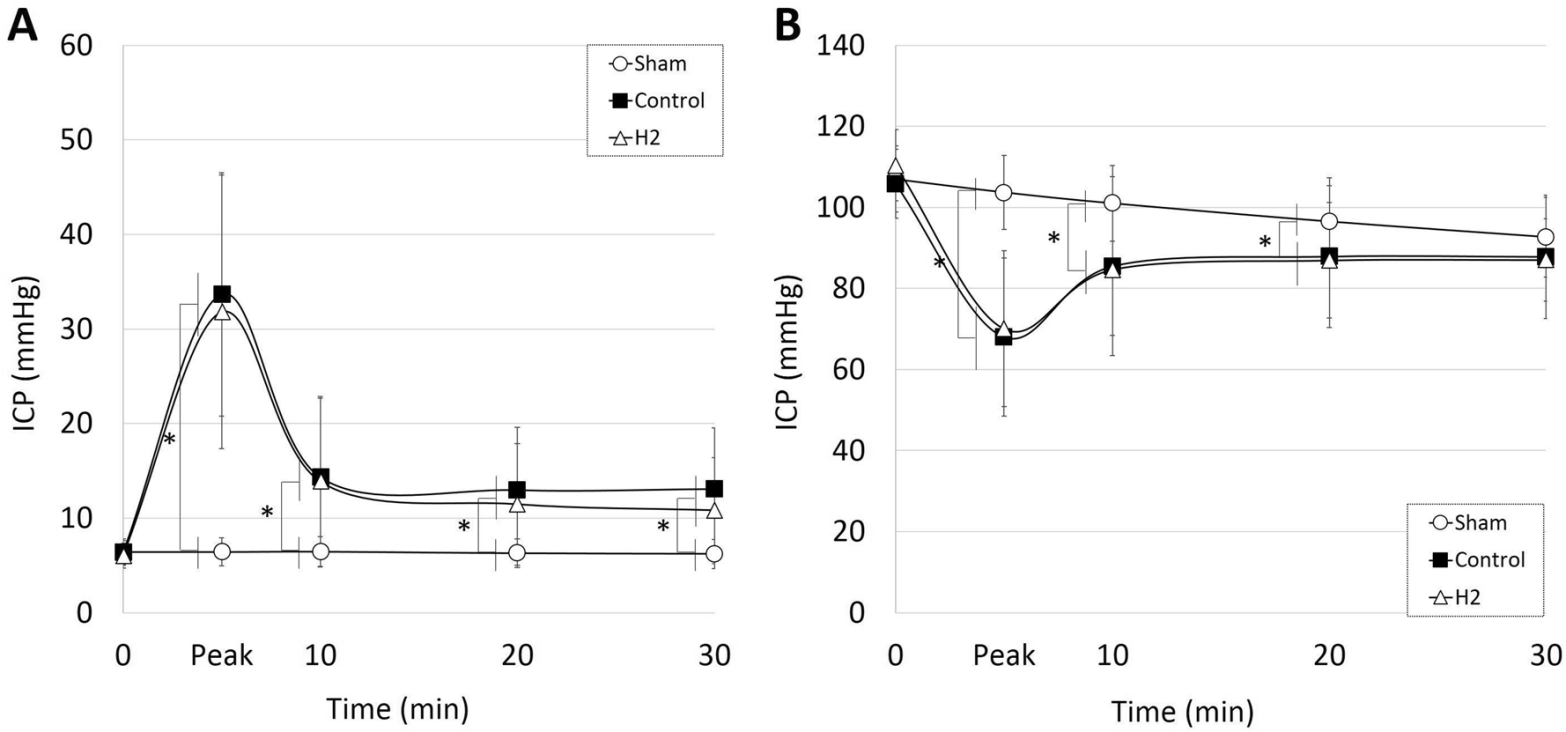
Time course of intracranial pressure (ICP) and cerebral perfusion pressure (CPP). A and B: ICP (**A**) and CPP (**B**) were recorded from subarachnoid hemorrhage (SAH) induction for the initial 30 min (n = 31 in sham group, n = 35 in control group, and n = 33 in H_2_ group). *P < 0.05.

Baseline ICP did not significantly differ between the three groups (n = 31 in sham group, n = 35 in control group, and n = 33 in H_2_ group; *P* > 0.05, respectively). Time course of ICP showed no significant difference between the control (n = 35) and H_2_ (n = 33) groups (at 0, peak, 10, 20 and 30 min; *P* > 0.05, respectively). ICP values in these two groups were significantly higher during the peak and initial 30 min of the experiment compared to the sham groups (*P* < 0.05, respectively) (Fig. 2A). MABP and CPP values in the control and H_2_ groups decreased sharply after SAH induction and approached the values of the sham group within 30 min. CPP demonstrated significantly lower values during the 10 min after SAH induction in the control and H_2_ groups compared to the sham group (*P* < 0.05, respectively) (Fig. 2B).

### Body weight loss and neurological deficits

**Figure 3:**
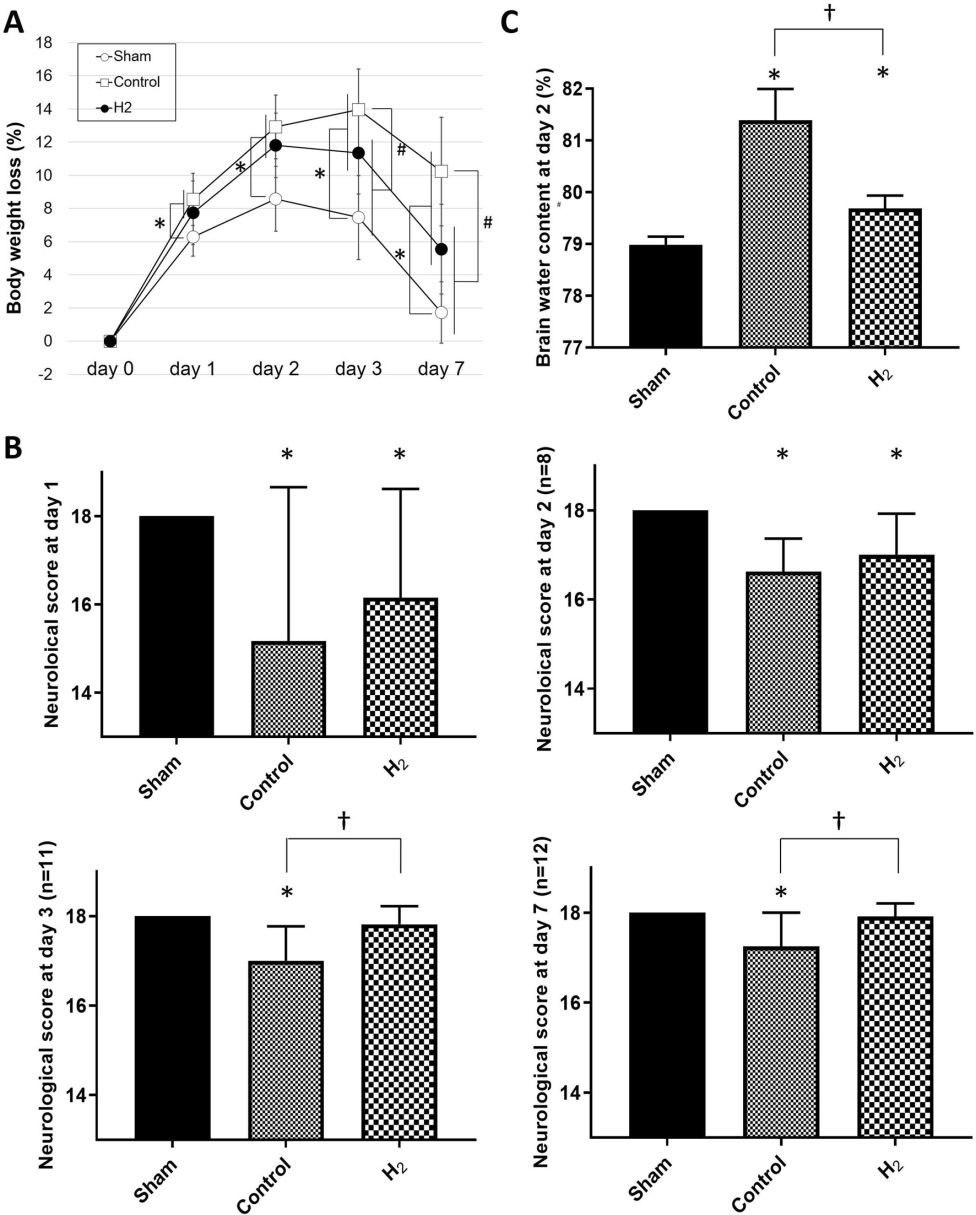
Body weight loss, neurological scores, and brain edema were significantly improved with H_2_ gas inhalation despite hypoperfusion. (**A**) Changes in the body weights of rats were measured on days 0, 1, 2, 3, and 7 after SAH. Rat numbers on day 0: n = 31 in sham group, n = 35 in control group, and n = 33 in H_2_ group; day 1: n = 31, day 2: n = 31, day 3: n = 23, and day 7: n = 12. *P < 0.05 compared with sham group, ^#^*P* < 0.05 compared with control group. (**B**) Neurological scores on days 1, 2, 3, and 7 are shown. Rat numbers on day 1: n = 31 in sham group, n = 35 in control group, and n = 33 in H_2_ group; day 2: n = 8, days 3: n = 11, and 7: n = 12. *P < 0.05. (**C**) Brain water content of cerebral cortex was measured on day 2. All groups: n = 5. *P < 0.05.

Body weight loss was significantly greater in the control and H_2_ groups compared to the sham group on day 1 (*P* < 0.05, respectively). Body weight loss was significantly lower in the H_2_ group compared to the control group on days 3 and 7 (*P* < 0.01, respectively) (Fig. 3A). Neurological score was significantly improved in the H_2_ group compared to the control group on days 3 and 7 (*P* < 0.01, respectively) (Fig. 3B).

### Brain water content

Brain water content showed significant increases in the control and H_2_ groups compared to the sham group on day 2 (*P* < 0.0001 and *P* < 0.05, respectively). Brain water content was significantly higher in the control group compared to the H_2_ group (*P* < 0.0001) (Fig. 3C).

### Expression of S100B and p-JNK

**Figure 4:**
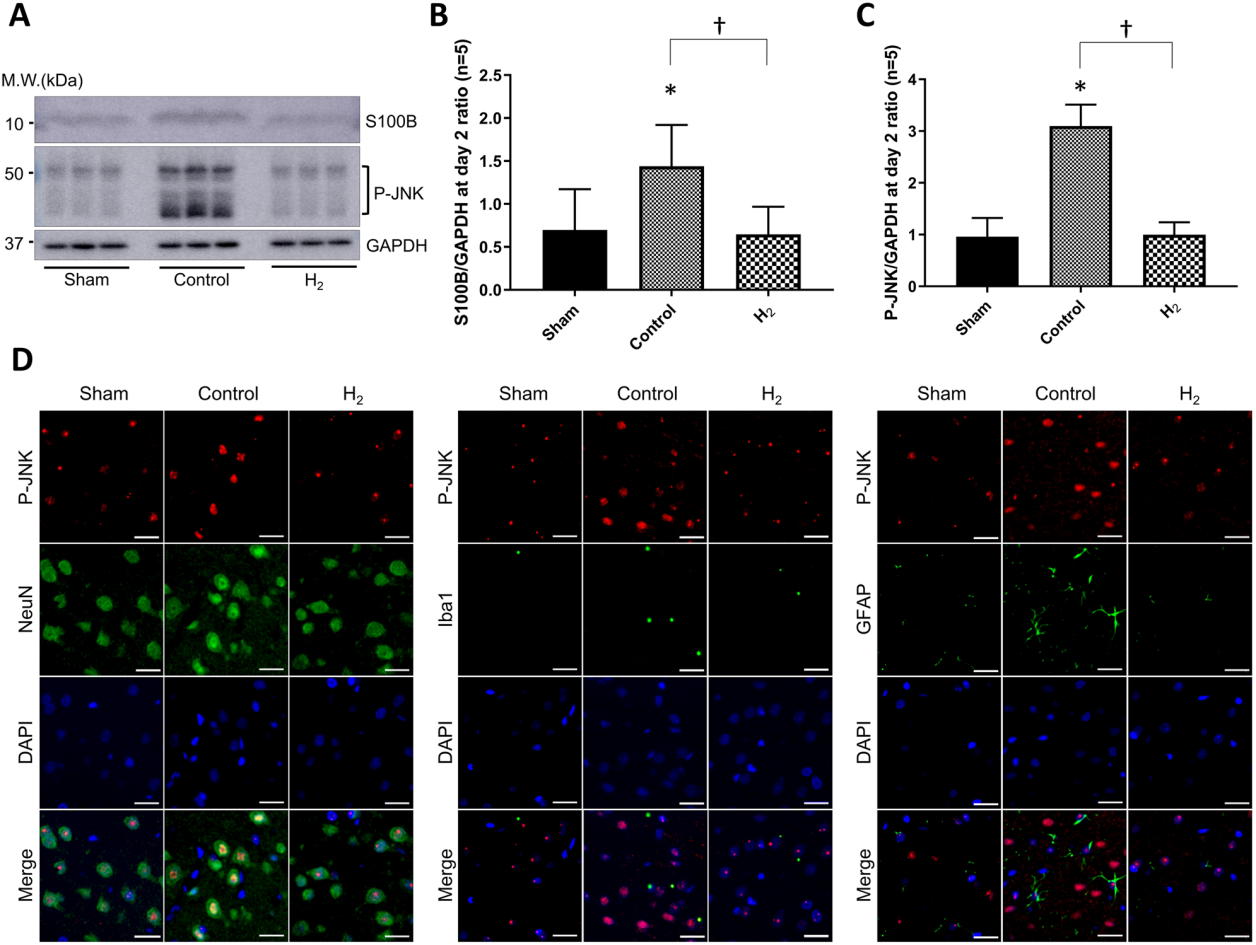
Expression of S100 calcium-binding protein B (S100B) and phosphorylation of C-Jun N-terminal kinase (p-JNK) in the ipsilateral cortex were significantly improved with H_2_ gas inhalation despite hypoperfusion. (**A**) Representative bands of S100B and p-JNK in the left temporal cortex together with glyceraldehyde-3-phosphate dehydrogenase (GAPDH) as a housekeeping protein (n = 5, representatively). (**B**,**C**) Quantification of S100B/GAPDH (**B**) and p-JNK/GAPDH (**C**). *P < 0.05. D: Representative images of immunofluorescence double staining of p-JNK (red) with neuronal nuclear antigen (NeuN), Iba1, or glial fibrillary acidic protein (GFAP) (green) in the left temporal cortex in the control group and H_2_ group. Nuclei are stained blue with 4′,6-diamidino-2-phenylindole. Scale bar = 20 μm.

Expression of S100B and p-JNK in the left temporal cortex were significantly lower in the H_2_ group compared to the control group at day 2 (*P* < 0.05 and *P* < 0.0001, respectively) (Fig. 4A–C). P-JNK was mainly expressed in the neurons and microglia, not astrocytes (Fig. 4D).

### Reactive astrogliosis

**Figure 5:**
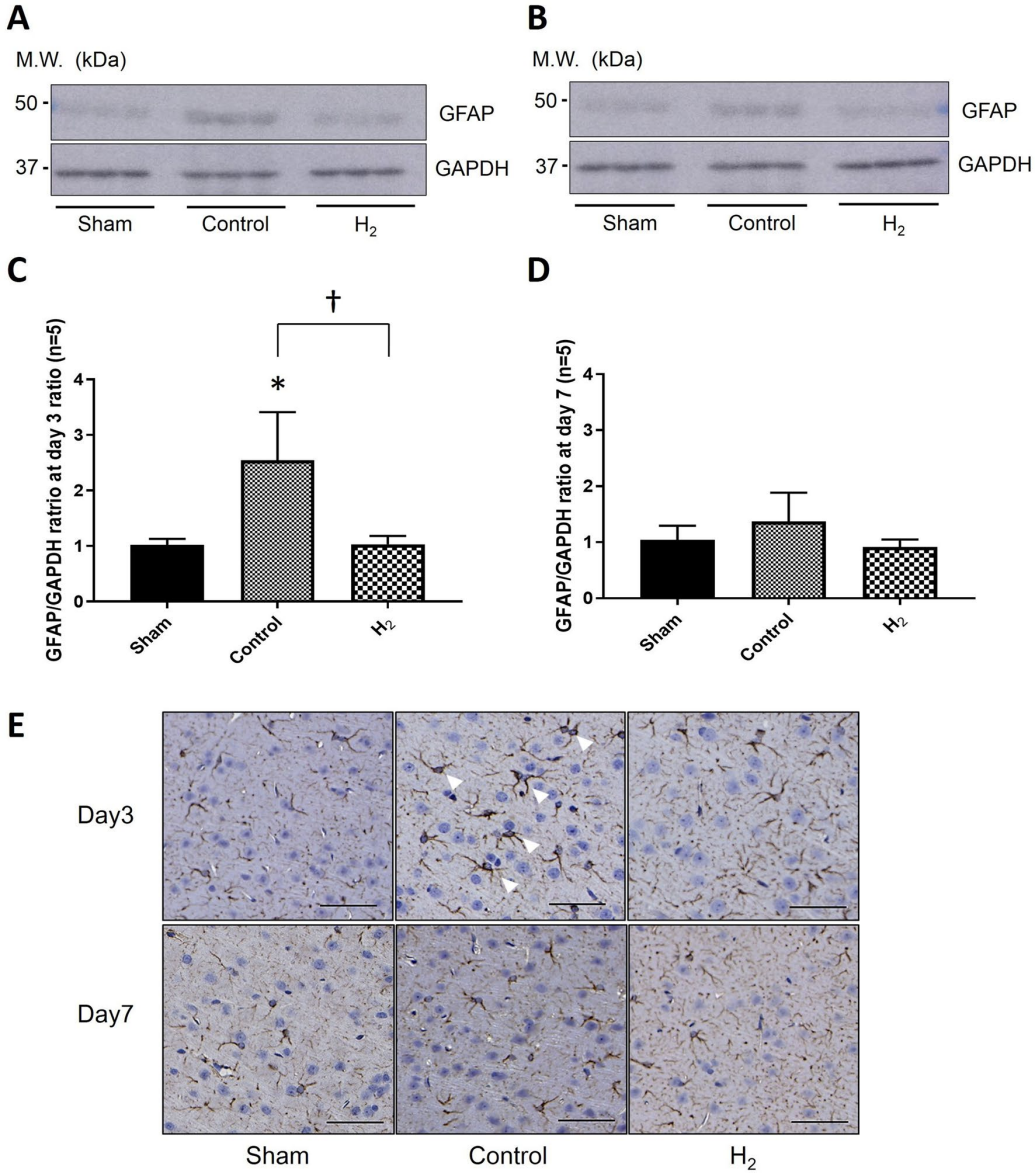
Reactive astrogliosis in the ipsilateral cortex was significantly improved with H_2_ gas inhalation despite hypoperfusion. (**A**,**B)** Representative bands of GFAP expression together with GAPDH as a housekeeping protein in the left temporal cortex are shown on days 3 (n = 6; **A**) and 7 (n = 7; **B**). (**C**,**D**) Graphs present quantification of the GFAP/GAPDH mean optical density ratio on days 3 (**C**) and 7 (**D**). (**E**) Representative microscopic images of GFAP-stained cross sections of the left temporal cortex on days 3 and 7 (scale bar = 50 μm). *P < 0.05.

GFAP expression showed a significant decrease in the H_2_ group compared to the control group on day 3 (*P* < 0.01) (Fig. 5A,C,E). However, no significant difference was found between all groups on day 7 (*P* > 0.05) (Fig. 5B,D,E).

### Analysis of CV in the distal ACA

**Figure 6:**
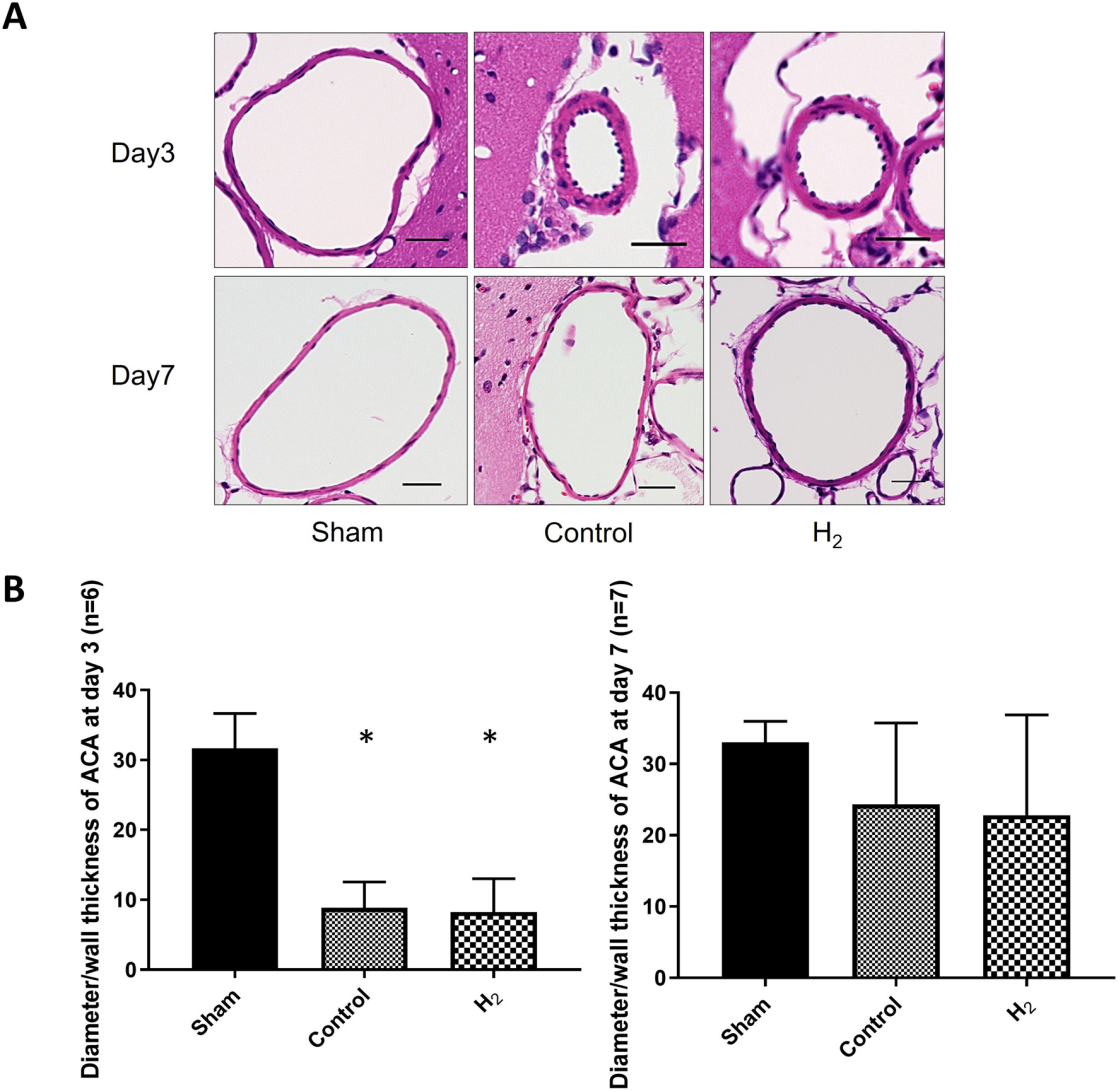
Cerebral vasospasm in the distal anterior cerebral artery (ACA) was not improved with hydrogen (H_2_) gas inhalation. (**A**) Representative microscopic images of hematoxylin and eosin-stained cross sections of the distal ACA are shown after 3 (n = 6) and 7 days (n = 7), (scale bar = 30 μm). (**B**) Inner diameter/wall thickness in the distal ACA on days 3 and 7. *P < 0.05.

CV in the distal ACA was significantly worsened in the control and H_2_ groups compared to the sham group on day 3 (*P* < 0.0001, respectively). CV showed no significant difference between the control and H_2_ groups (*P* = 0.9715). CV had improved in the control and H_2_ groups on day 7, with no significant difference between all groups (*P* = 0.1790) (Fig. 6).

### Nissl staining in the left temporal lobe and DG

**Figure 7:**
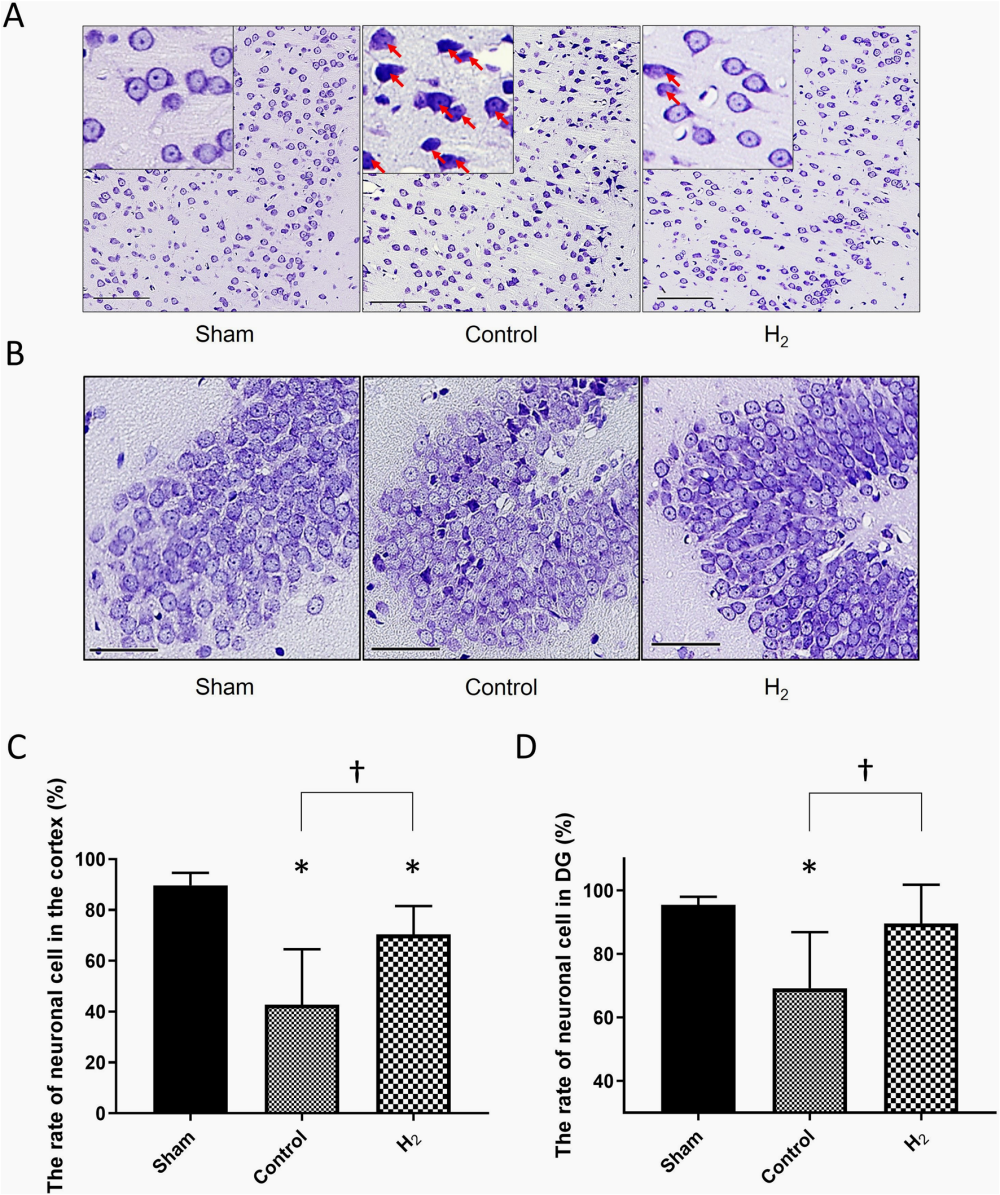
Neuronal cell death in the ipsilateral cortex and dentate gyrus (DG) was significantly improved with H_2_ gas inhalation despite hypoperfusion. (**A**,**B**) Representative microscopic images of Nissl-stained cross section of the left temporal cortex (**A**) and DG (**B**) are shown after 7 days (n = 7, scale bar = 100 μm and 50 μm, representatively). The boxed region of (**A**) demonstrates magnified images. Arrowheads demonstrate dead neuronal cells. (**C**,**D**) Numbers of living neuronal cells in the images of A (**C**) and B (**D**) were measured and calculated. *P < 0.05.

The numbers of living neuronal cells in the left temporal lobe and DG were decreased significantly less in the H_2_ group compared to the control group (*P* < 0.001, and *P* < 0.05, respectively) (Fig. 7).

## Discussion

The present study showed that 1.3% H_2_ gas inhalation ameliorated DBI through the effect of reducing EBI but without improving CV in an experimental SAH rat model. DBI manifesting as focal neurological deficits and/or cognitive deficits is considered to be the most important cause of mortality and morbidity after SAH^2^. DBI has multiple causes such as exacerbation of EBI, reactive astrogliosis, and/or CV^2,20^.

EBI may be associated with several events including increased ICP, decreased cerebral blood flow, and global cerebral ischemia occurring after SAH, and may result in blood–brain barrier disruption, and inflammation leading to brain edema and neuronal cell death^21^. S100B, ROS, and JNK are very important in the pathophysiological changes occurring in EBI^21–23^. S100B is a Ca^2+^-binding protein mainly produced and released by astrocytes^23,24^. JNK is one of the mitogen-activated protein kinases. S100B secretion from astrocyte increases after brain injury, resulting in increased ROS in astrocytes, microglia, and neurons. Increased ROS induces p-JNK in neurons and induces apoptosis. Enhanced S100B release transforms astrocytes into reactive astrocytes characterized by up-regulation of GFAP and S100B. Enhanced p-JNK transforms microglia into reactive microglia. Consequently, reducing ROS or p-JNK improves outcomes after SAH^7,25^.

H_2_ has strong antioxidant activity and high tissue transferability, and can be safely administered to live animals and patients. The potent antioxidant effect of H_2_ is mediated by selective inhibition of highly toxic ROS such as hydroxyl radical (OH·) and peroxynitrite (ONOO^−^)^6^. H_2_ gas inhalation ameliorates EBI after SAH by suppressing ROS*,*^7^ but the mechanism was not investigated and no effect on DBI was shown.

In the present study, 1.3% H_2_ gas treatment was performed only on days 0 and 1 of EBI after SAH. H_2_ gas inhalation ameliorated the effects of EBI including brain water content, expression of S100B and p-JNK, and reactive astrogliosis. Such amelioration of EBI is expected to result in reduced DBI including better neurological state and reduced neuronal cell damage but without improvement of CV.

The different findings of these two studies can be attributed to differences in the experimental models and treatment periods. The present study used the SAH + UCCAO model, whereas the previous study^7^ used a general high mortality EVP model.

The EVP model is considered to be the best model for human SAH, but is unsuitable for studying DBI because of lack of control of the hemorrhage and high mortality rate. In contrast, our SAH + UCCAO model is a modified EVP model with lower mortality rate, which is more suitable for the observation of EBI, CV, and DBI^12^. Recent clinical studies have revealed that early cerebral hypoperfusion within 3 days after SAH is very important to prevent the occurrence of CV and DBI. Consequently, the present study could assess the effectiveness of H_2_ gas against DBI as well as EBI. The SAH + UCCAO model mimics the clinical course of early cerebral hypoperfusion after SAH by inducing early cerebral hypoperfusion in the mild SAH EVP model with lower ICP by performing UCCAO at 24 h after induction of SAH.

In this study, 1.3% H_2_ gas inhalation was performed on days 0 and 1, starting before SAH onset and continuing for 2 h on day 0, and starting before addition of UCCAO and continuing for 30 min on day 1. In the previous study^7^, H_2_ gas inhalation was performed for 2 h beginning at 1 h after SAH. The best time point for H_2_ treatment might be 30 min before the experiment because the concentration of H_2_ in the rat brain peaks at 30 min after the start of H_2_ gas inhalation^26^. Pre-treatment with H_2_ is also effective in rat models of cerebral ischemia reperfusion injury and rhabdomyolysis-induced acute kidney injury^27,28^. Interestingly, the infarct volume was significantly decreased in the rat ischemia model only after H_2_ gas inhalation during reperfusion, not during ischemia^6^. The findings suggest that inhalation of H_2_ gas starting before endovascular coiling surgery for ruptured or unruptured cerebral aneurysm might reduce brain damage even if rerupture of the cerebral aneurysm or SAH occurs during surgery. In addition, since rerupture of cerebral aneurysm is a risk in patients with SAH, immediate inhalation of H_2_ gas after diagnosis of SAH might reduce the brain damage caused by rerupture. Further investigations are needed to optimize the timing and duration of H_2_ gas inhalation.

The present study found no significant improvement in CV after 1.3% H_2_ gas inhalation, possibly because H_2_ gas administration had been stopped before CV occurred. However, hemoglobin leakage from the blood vessels is known to occur after SAH induces production of ROS^29^. CV is improved by reduction of p-JNK or administration of hydrogen-rich saline^8,30^, and CV may be reduced if H_2_ gas is inhaled for a long time after SAH. Further study will be needed to elucidate the effect of H_2_ gas on CV.

H_2_ gas inhalation induces almost no side effects. In the future, in addition to inhalation of H_2_ gas before coiling surgery, inhalation of H_2_ gas in patients with suspected SAH in the ambulance before arrival at the hospital might reduce the brain damage caused by SAH. Further investigations are needed to assess the potential effects of H_2_ after SAH occurrence within the limitations of actual clinical practice.

The present study has some limitations. First, traumatic complications were induced in the contralateral cortex by application of the ICP probe. This is a well-known disadvantage of ICP probe insertion between the dura mater and skull in the rat model, and the possibility of damage in other areas of the brain cannot be denied^31^. Long-term functional impairments after SAH were not evaluated for more thorough assessment of delayed ischemic neurological deficits.

## Supplementary information

Supplementary file 1 (DOCX 944 kb)
