# Supplementary material for: Hydrogen gas inhalation improves delayed brain injury by alleviating early brain injury after experimental subarachnoid hemorrhage
Source: Sci Rep. 2020 Jul 23;10:12319. doi: 10.1038/s41598-020-69028-5 (PMC7378202; doi:10.1038/s41598-020-69028-5)

**Hydrogen Gas Inhalation Improves Delayed Brain Injury by Alleviating Early Brain Injury After Experimental Subarachnoid Hemorrhage**

Kosuke Kumagai, MD*^1^; Terushige Toyooka, MD, PhD^1^; Satoru Takeuchi, MD, PhD^1^; Naoki Otani, MD, PhD^1^; Kojiro Wada, MD, PhD^1^; Arata Tomiyama, MD, PhD^1^; Kentaro Mori, MD, PhD^2^

^1^Department of Neurosurgery, National Defense Medical College, Tokorozawa, Saitama, Japan; ^2^Department of Neurosurgery, Tokyo General Hospital, Tokyo, Japan

Corresponding author: Kosuke Kumagai, MD, Department of Neurosurgery, National Defense Medical College, 3-2 Namiki, Tokorozawa, Saitama 359-8513, Japan.

E-mail: kumagaikousuke0714@yahoo.co.jp

Tel: +81-4-2995-1511

Fax: +81-4-2996-5207

Cover title: H_2_ Inhalation Improves DBI After Experimental SAH

Tables: 0, Figures: 7

**Key Words:** subarachnoid hemorrhage, endovascular perforation model, delayed brain injury, hydrogen

Raw data of Western Blot

Figure 4.


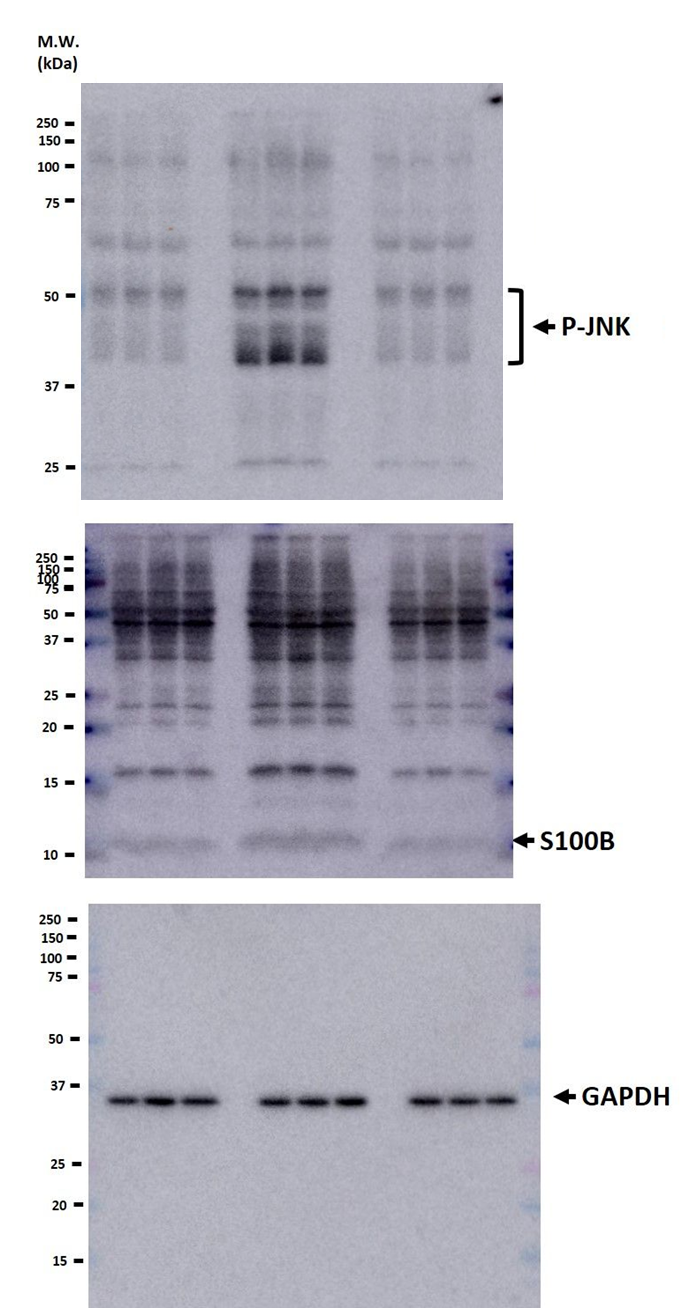


Figure 5.


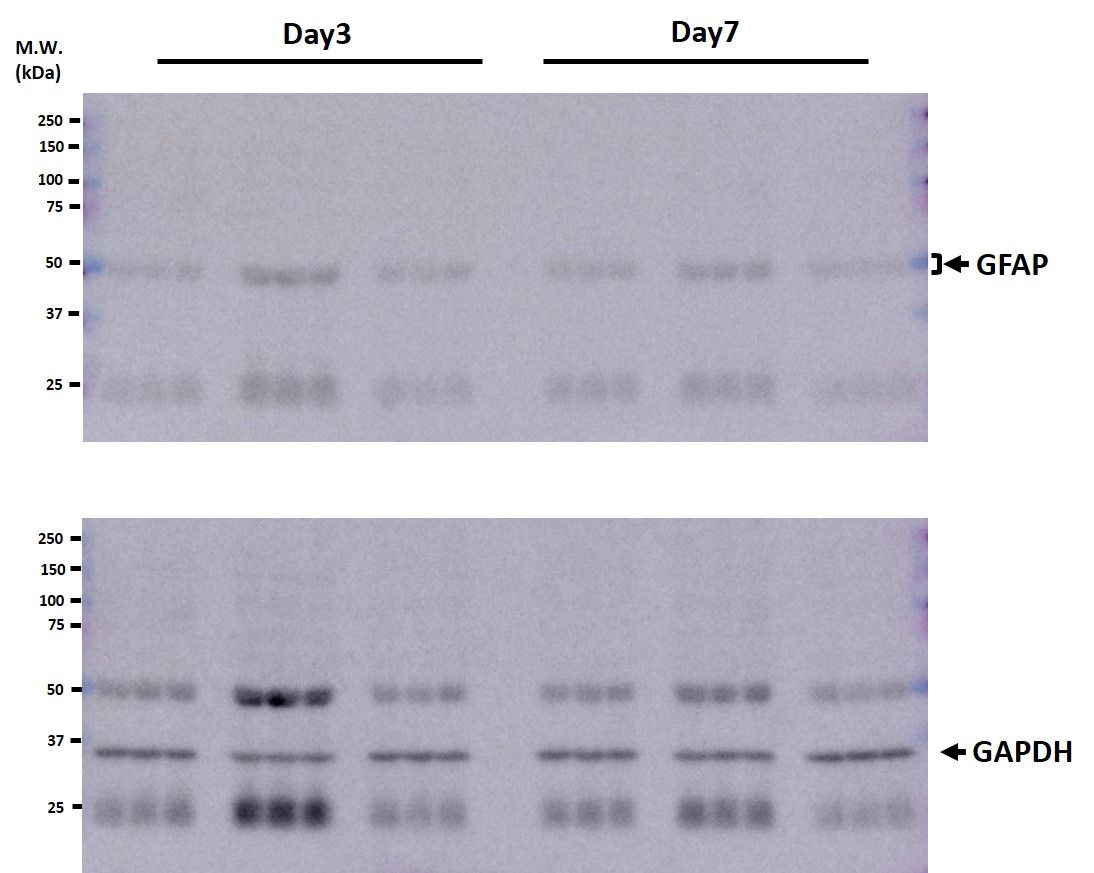

Supplement: Supplementary file 1 — (DOCX 944 kb) [file 41598_2020_69028_MOESM1_ESM.docx]
